# The Effect of *Lupinus albus* and Calcium Chloride on Growth Performance, Body Composition, Plasma Biochemistry and Meat Quality of Male Pigs Immunized Against Gonadotrophin Releasing Factor

**DOI:** 10.3390/ani6120078

**Published:** 2016-12-01

**Authors:** Karen Moore, Bruce Mullan, Jae Cheol Kim, Frank Dunshea

**Affiliations:** 1 Faculty of Veterinary and Agricultural Sciences, The University of Melbourne, Parkville, VIC 3010, Australia; fdunshea@unibmelb.edu.au; 2 Grains and Livestock Industries, Department of Agriculture and Food Western Australia, South Perth, WA 6151, Australia; bruce.mullan@agric.wa.gov.au (B.M.); jae.kim@abvista.com (J.C.K.)

**Keywords:** albus lupins, calcium chloride, fat deposition, growth performance, immunocastrated male pigs, objective meat quality

## Abstract

**Simple Summary:**

Pigs immunized against gonadotrophin releasing factor (immunocastrated (IC) males) have an increased feed intake, growth rate, back fat and fat deposition compared to entire males. It is desirable to develop management strategies to limit the increase in feed intake and fat deposition in IC males. This experiment used in-feed ingredients (*Lupinus albus* (albus lupins) or a combination of calcium chloride and sodium tri-polyphosphate (mineral salts)) to try to suppress the voluntary feed intake of IC male pigs and subsequently fat deposition. Mineral salts decreased feed intake with no effect on fat deposition while albus lupins reduced both feed intake and fat deposition in pigs.

**Abstract:**

Two hundred and ninety-four pigs were used to assess the effect of two ingredients (*Lupinus albus* (albus lupins) or a combination of calcium chloride and sodium tri-polyphosphate (mineral salts)) on growth performance, body composition and objective meat quality of pigs immunized against gonadotrophin releasing factor (immunocastrates) and entire male pigs in the late finishing phase. Pigs fed mineral salts ate less feed than those fed the control diet with no effect on growth rate (*p* > 0.05), backfat (*p* > 0.05) or fat deposition (*p* > 0.05). Pigs fed albus lupins had a reduced feed intake (*p* < 0.001 for all time periods), lower growth rate (*p* < 0.001 for all time periods), lower backfat (*p* < 0.005) and decreased fat deposition (*p* < 0.001 for all time periods) compared to those fed the control diet or mineral salts. From day (d) 0–28 pigs fed mineral salts had a better feed conversion ratio (*p* = 0.001) than those fed albus lupins who in turn had an improved feed conversion compared to the control diet. Immunocastrates had thicker backfat than entire males at the end of the experiment (*p* < 0.001), however, feeding albus lupins to immunocastrated males reduced backfat thickness to similar to entire males fed the control diet (*p* = 0.01). With the exception of the increased muscle pH at 45 min post-exsanguination in mineral salts and albus lupins compared with the control diet (*p* = 0.03) there was no effect of diet on objective pork quality. Pork from IC males had a higher ultimate pH (*p* < 0.001), was lighter (L*; *p* = 0.003), more yellow (*p* = 0.008) and had a higher drip loss (*p* < 0.001) compared to entire males. Albus lupins show potential in reducing the increase in feed intake and backfat associated with immunocastration. Mineral salts may be useful in situations where a reduction in feed intake and an improvement in feed conversion is desired and reducing fat deposition is not the objective.

## 1. Introduction

Immunization of entire males against gonadotrophin releasing factor (GnRF) is an effective strategy to eliminate boar taint and a welfare friendly alternative to physical castration [1]. It involves two immunizations with an incomplete analogue of GnRF conjugated to a carrier protein in a low reactogenic-adjuvant system [2]. Following the second immunization the pig has an increased feed intake and growth rate but there is also an increase in backfat and whole body fat deposition compared to entire males [2,3,4,5,6,7,8]. Management strategies to limit the increase in feed intake and fat deposition and promote lean deposition are required for markets where producers are penalized for high backfats.

A way to reduce the increase in feed intake and increase in fat in immunocastrated (IC) male pigs is to restrict feed intake as restrictively fed IC male pigs have a reduced backfat [9] and increased carcass leanness [10]. However, restricting feed intake by restricting the amount of feed in a group-housed environment has welfare issues in terms of increased aggression [10]. If a feeding strategy can be identified that reduces feed intake of IC males to similar to that of entire males, then this could be a sound management strategy to limit the increase in feed intake and fat deposition associated with the production of IC male pigs.

The aim of this experiment was to use feed ingredients such as *Lupinus albus* (albus lupins) or a combination of calcium chloride and sodium tri-phosphate to suppress the voluntary feed intake of IC male pigs when fed ad libitum. The possible effect of these ingredients on objective meat quality was also explored to ensure that the meat quality was not negatively impacted. Albus lupins have been found to reduce feed intake in several pig experiments [11,12]. Dunshea et al. [11] suggested that the most likely mechanism by which albus lupins affect feed intake is by delayed transit in the stomach and small intestine. This delayed transit then feedbacks on satiety signals. A combination of calcium chloride and sodium tri-phosphate (mineral salts) has reduced feed intake in the range of 6% to 15% [13,14]. It suppresses the pig’s appetite by changing the acid to base ratio by increasing the plasma chloride level and subsequently creating a HCO_3_^−^ deficiency [13].

The hypotheses tested in this study were (1) pigs immunized against GnRF which are fed either a diet containing albus lupins or mineral salts will have a reduced feed intake with no effect on growth rate compared to pigs receiving a standard finisher diet; (2) pigs fed either albus lupins or mineral salts will deposit less fat compared to pigs receiving a standard finisher diet; (3) pigs immunized against GnRF and fed either a diet containing albus lupins or mineral salts will have a similar backfat compared to entire males receiving a standard finisher diet and; (4) feeding pigs a diet containing albus lupins or mineral salts will have no effect on objective meat quality compared to a standard finisher diet.

## 2. Materials and Methods

The experimental protocol used was approved by the Department of Agriculture and Food Western Australia’s Animal Research Committee and by the Animal Ethics Committee (activity number 1-15-02). The animals were handled according to the Australian code of practice for the care and use of animals for scientific purposes [15]. A total of 294 Large White × Landrace × Duroc entire male and immunocastrated male pigs were used in this experiment. The experiment was a 2 × 3 factorial with the main treatments being: (i) sex and lysine concentration (sex; entire males fed a diet with 0.64 g standardized ileal digestible (SID) lysine/MJ DE (mega joule digestible energy) for four weeks (entire males) or IC males fed a diet with 0.64 g SID lysine/MJ DE for two weeks followed by 0.50 g SID lysine/MJ DE for two weeks (IC male)); and (ii) feed ingredient (diet; control, 3% calcium chloride + 1.6% sodium tripolyphosphate, or 300 g/kg albus lupins).

### 2.1. Allocation and Housing

Pigs were sourced from a high health status commercial herd at 34.9 ± 3.98 kg (mean ± SD) liveweight (LW). Upon arrival pigs were individually identified with ear tags, weighed and stratified on their LW. The allocated pigs received a priming dose of anti-gonadotrophin releasing factor immunological product (Improvac^®^, Zoetis Australia, Rhodes, Australia) on d −28 (where d 0 is when all pigs received the second dose of the anti-gonadotrophin releasing factor vaccine). Pigs were group housed (*n* = 7) in a naturally ventilated grower shed. They had ad libitum access to water, and a commercial feed via a single spaced feeder.

### 2.2. Diets and Feeding Regime

On d 0 all pigs received the experimental diet and the second dose of the anti-gonadotrophin releasing factor vaccine was given to the allocated pigs who had received the priming dose on d −28. The entire males did not receive a placebo injection. The experimental diets were formulated to the same nutrient specifications (14 MJ DE and 0.64 g SID lysine/MJ DE (high) or 0.50 g SID lysine/MJ DE (low)). The diets were formulated so that the IC male pigs were fed as entire males for 2 weeks (from d 0; high) and then the lysine level in the diet was reduced for the remaining 2 weeks (low; based on recommendations from Moore et al. [7]). The entire male pigs continued to receive the diet adequate for an entire male pig (high). The composition of the experimental diets is given in Table 1. The diets were also analyzed for quantitative amino acid composition (Australian Proteome Analysis Facility, Sydney, Australia) and the results are presented in Table 2.

### 2.3. Growth Performance

Pigs were weighed weekly and feed refusals determined on d 0, 7, 14, 21 and 28 to measure average daily gain and voluntary feed intake. The feed conversion ratio was calculated on a weekly basis from when the feeding of the experimental diets commenced.

### 2.4. Dual-Energy X-ray Absorptiometry Analysis

Twelve pigs per treatment (3 pigs/pen randomly selected from 4 replicate pens, so 72 in total (12 pigs × 6 treatments)) were scanned on d −1, 13 and 27 using dual-energy x-ray absorptiometry (DXA). The pigs were removed from feed and fasted for approximately 16 h before scanning. Immediately before scanning the pigs were weighed and then transferred to the DXA facility. They were injected intramuscularly with Stresnil^®^ (azaperone 40 mg/mL, Stresnil Neuroleptic Injection for Pigs, Ausrichter Pty Ltd., Newtown, Australia) at 2 mL/10 kg LW. When sufficiently sedated the pigs were transferred to the DXA machine (Norland XR46 Densitometer Machine, Norland Products Inc., Cranbury, NJ, USA) [16]. The pigs were scanned in ventral-recumbency, with hind legs extended and forelegs positioned caudally. Whole body mode was used to scan and the scan was subsequently analyzed using whole body analysis. Measurements made by DXA included lean tissue mass, fat tissue mass and bone mineral content. After scanning the pigs were placed in a recovery room until they were able to stand and were then returned to their pens. The pigs were given their respective diets on return to their individual pens.

### 2.5. Slaughter Procedure

Four weeks after the diets were introduced pigs were individually tattooed, removed from feed overnight and transported to a commercial abattoir (approximately 90 min transport time). The pigs were stunned using a carbon dioxide, dip-lift stunner set at 85% CO_2_ for 1.8 min (Butina, Holbæk, Denmark). Exsanguination, scalding, dehairing and evisceration were performed using standard commercial procedures. Hot carcass weight (HCW, AUSMEAT Trim 13; head off, fore trotters off, hind trotters on; AUS-MEAT Ltd., South Brisbane, Australia) and P2 backfat depth, 65 mm from the dorsal midline at the point of the last rib (PorkScan Pty Ltd., Canberra, Australia) were measured approximately 35 min after exsanguination, prior to chiller entry (2 °C, airspeed 4 m/s).

### 2.6. Blood Analysis

Blood samples (20 mL in lithium heparin tubes) were collected on d 0, 7, 14, 21 and 28 from the same pigs that were selected for DXA scanning. The blood samples were centrifuged at 2000× *g* for 15 min to recover plasma and were stored at −20 °C until analysed. Plasma urea nitrogen (PUN) was quantified using a commercial kit (Beckman Coulter/Olympus Reagent Kit Cat. No. OSR6134 Lot #6042). Plasma urea (mmol/L) was converted to PUN (mg/dL) by dividing by 0.357. Calcium was quantified using a commercial kit (Beckman Coulter/Olympus Reagent Kit Cat. No. OSR60117 Lot #6564). Phosphorus was quantified using a commercial kit (Beckman Coulter/Olympus Reagent Kit Cat. No. OSR6122 Lot #5797). Glucose was quantified using a commercial kit (Beckman Coulter/Olympus Reagent Kit Cat. No. OSR6121 Lot #5512). The assays for PUN, calcium, phosphorus and glucose were performed on an automated analyzer according to the manufacturer’s instructions (Olympus AU400; Olympus UK Ltd., Hertfordshire, UK). Plasma leptin was determined using a commercial ELISA kit (Cusabio Pig Leptin ELISA (CSB-E06815p, Jomar Life Research Pty Ltd., VIC, Australia). Sodium and chloride were measured by indirect ion selective electrodes and the assays were performed on an automated analyzer (AU680, Beckman Coulter, Sydney, Australia). Total carbon dioxide was determined by reaction with phophoenolpyruvate carboxylase reagent and the assay was performed on an automated analyzer (AU680, Beckman Coulter, Sydney, Australia).

### 2.7. Objective Meat Quality

Twenty-one pigs per treatment (3 pigs/pen that were DXA’d (from 4 replicate pens) and an additional 3 pigs/pen which were randomly selected from the remaining 3 replicate pens) were used to assess meat quality. pH and temperature decline in the *Longissimus thoracis* (LT) was measured at 45 min post-exsanguination using a portable pH/temperature meter (Cyberscan pH 300, Eutech Instruments, Singapore) fitted with a polypropylene spear-type gel electrode (Ionode IJ44, Ionode Pty Ltd., Brisbane, Australia) and a temperature probe. The pH meter was calibrated on two standards (pH 4.01 and 7.0) as per the manufacturer’s instructions. At 24 h post-slaughter a section of the LT muscle was removed from the left hand side of the carcass between the 12th and 13th rib. For determination of pH and temperature a 2 cm steak was cut from the appropriate sample and measured using the pH/temperature meter as previously described. Drip loss was measured using a modification of the method described by Rasmussen and Andersson [17]. The muscle was cut to a 50 g cube then wrapped in netting and suspended in a sealed plastic container. The samples were stored for 24 h at 4 °C. The sample was then removed and gently patted dry to remove excess moisture before being re-weighed. Colour (L*, a* and b*) was measured with a Minolta Chromameter CR-400 (Minolta, Osaka, Japan), using D65 illumination, a 2° standard observer, and an 8-mm aperture in the measuring head, standardized to a white tile after a bloom time of 30 min (determined from the freshly cut 2 cm steak). An 80 ± 10 g (approximately 5 cm × 2 cm × 2 cm) sample was cut from the loin samples to measure cooking loss and shear force [18]. The samples were frozen in individual bags. The bagged frozen samples were then suspended from a metal rack and placed in a water bath which had been pre-heated to 70 °C. The samples were cooked at 70 °C until an internal temperature of 70 °C was reached (approximately 30 min). After removal from the water bath, the samples were allowed to cool in iced water for 30 min, patted dry to remove excess moisture, and re-weighed before being refrigerated at 4 °C overnight. Cooking loss percentage for each sample was determined by dividing the difference in the raw and cooked weights by the weight of the raw pork sample. The cooked sample was then cut into five cross-section samples (1 cm^2^) parallel to the muscle fibres. Warner Bratzler shear force was measured using a Warner Bratzler shear blade fitted to a Lloyd Texture Analyzer (TA-2, Lloyd Instruments Ltd., Bognor Regis, UK).

### 2.8. Statistical Analysis

General analysis of variance was performed with the GENSTAT 16 program (VSN International Ltd., Hemel Hempstead, UK) to analyze the main effects of sex and lysine concentration and feed ingredient on growth performance, carcass quality, body composition, physiological measures and objective pork quality measures. For growth performance, and carcass data, the pen was the experimental unit. For the DXA, physiological measures and objective pork quality measures, pig was the experimental unit. Repeated measures analysis of variance was used to analyze the blood measures. Batch was used as a block in the analysis. A level of probability of less than 0.05 was used to determine statistical difference between the means. A level of probability of less than 0.1 but greater than 0.05 was determined to be a trend. Fisher’s-protected least significant differences were used to determine differences among treatments.

## 3. Results

There were acceptance issues in the albus lupin diet with the inclusion of albus lupins at 30%. After the first week the inclusion rate of albus lupins was reduced to 15% for one week to allow the pigs to compensate and then increased to 20% for the remaining two weeks. Following the dilution of the albus lupin diet there were no issues associated with eating the albus lupins.

### 3.1. Growth and Carcass Performance

The growth and carcass performance results are presented in Table 3. Daily gain was not affected by sex from d 0 to 14 (*p* > 0.05). However, from d 15 to 28 and d 0 to 28 IC males grew faster compared to entire males (*p* < 0.001 for both). Immunocastrated males had an increased feed intake for d 0 to 14, d 15 to 28 and d 0 to 28 (*p* = 0.05, *p* < 0.001 and *p* < 0.001, respectively). The feed conversion ratio was not affected by sex from d 0 to 14 (*p* > 0.05). However, from d 15 to 28 and d 0 to 28 IC males had a worse feed conversion ratio compared to entire males (*p* < 0.001 for both). There was a trend for IC males to have a heavier carcass weight compared to entire males (*p* = 0.06). Immunocastrated males had a lower dressing percentage (*p* < 0.001) and a higher P2 backfat (*p* < 0.001) compared to entire males.

Irrespective of sex, pigs fed albus lupins had a lower daily gain compared to those fed the control or mineral salt diet for d 0 to 14, d 15 to 28 and d 0 to 28 (*p* < 0.001 for all) and there was no difference in average daily gain between the control diet and mineral salt diet. Pigs fed albus lupins ate less feed than those on the mineral salt diet who in turn ate less than those on the control diet for all time periods (*p* < 0.001, for all). From d 0 to 14 pigs fed the mineral salt diet had a better feed conversion compared to the other diets (*p* = 0.05). From d 15 to 28 pigs fed the albus lupin diet and the mineral salt diet had a better feed conversion than those fed the control diet (*p* = 0.007). From d 0 to 28 pigs fed the mineral salt diet had a better feed conversion than those on the albus lupin diet who in turn had a better feed conversion than the control diet (*p* = 0.001). Pigs fed the albus lupin diet had a lower carcass weight (*p* < 0.001), lower dressing percentage (*p* = 0.008) and a lower P2 backfat (*p* < 0.001) compared to those on the mineral salt and control diet.

There were no interactions (*p* > 0.05) between sex and feed for any growth or carcass measurement.

### 3.2. Body Composition

The body composition results are presented in Table 4. Bone mass composition was increased for IC males compared to entire males for d −1 to 13 (*p* = 0.03) and decreased from d 14 to 27 (*p* = 0.02). There was no difference between IC males and entire males for bone mass composition from d −1 to 27 (*p* > 0.05). There was no difference (*p* > 0.05) in lean deposition between IC males and entire males for all time periods. There was no difference (*p* > 0.05) in fat deposition between IC males and entire males for d −1 to 13. Immunocastrated males deposited more fat than entire males from d 14 to 27 (*p* < 0.001) and from d −1 to 27 (*p* < 0.001). Immunocastrated males also had an increased g fat per kg metabolic body weight from d 14 to 27 (*p* = < 0.001) and d −1 to 27 (*p* < 0.001) compared to entire males. There was no difference (*p* > 0.05) between sexes from d −1 to 13.

Pigs fed albus lupins had a lower bone mineral content than those fed a control or mineral salt diet from d −1 to 13 (*p* < 0.001). Pigs fed the mineral salt diet had a higher bone mineral content than those fed a control or albus lupin diet from d 14 to 27 (*p* = 0.003). From d −1 to 27 pigs fed albus lupins had a lower bone mineral content compared to those fed a control diet which in turn was lower than those fed the mineral salt diet (*p* < 0.001). Pigs fed albus lupins deposited less lean from d −1 to 13 (*p* < 0.001) and d 1 to 27 (*p* < 0.001) than those on a control or mineral salt diet. There was no difference between diets (*p* > 0.05) for d 14 to 27. Pigs fed albus lupins deposited less fat and had a lower g fat per kg metabolic body weight from d −1 to 13 (*p* < 0.001 for both), d 14 to 27 (*p* < 0.001 and *p* = 0.03, respectively) and d −1 to 27 (*p* < 0.001 for both) than those on a control or mineral salt diet.

There were no interactions (*p* > 0.05) between sex and feed for any body composition measurement.

### 3.3. Physiological Measures

**Figure 1:**
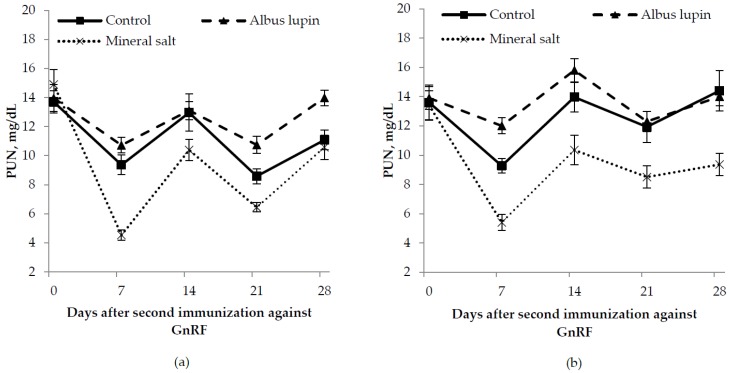
Change in plasma urea nitrogen (PUN) concentration (mean ± SE) for (**a**) entire males and (**b**) immunocastrated male pigs fed three different diets for 28 days after the second immunization against gonadotrophin releasing factor (GnRF) (*n* = 12). The *p*-values for time × sex, time × diet, time × sex × diet, sex, diet and time were *p* = 0.002, *p* < 0.001, *p* = 0.02, *p* = 0.07, *p* < 0.001 and *p* < 0.001, respectively.

There was a time by sex interaction (*p* = 0.002) for PUN concentration with PUN concentration on d 21 for entire males being less than IC males on d 21 (Figure 1). There was a time by diet interaction in that pigs fed the mineral salt diet had a lower PUN concentration on d 7 and d 14 compared to those on the control and albus lupin diets (*p* < 0.001). There was also a time by sex by diet interaction (*p* = 0.02) where IC male pigs fed the control diet on d 21 and 28 had a higher PUN concentration compared to entire male pigs fed the control diet. There was no difference in PUN concentration between sexes for the other diets. There was a trend for IC males to have a higher PUN concentration than entire males (*p* = 0.07). Pigs fed the mineral salt diet had a lower PUN concentration than those fed the control which in turn was lower than those fed the albus lupin diet (*p* < 0.001). Plasma urea nitrogen concentration varied with time. In general it was lower on d 7 and d 21 compared to the other days (*p* < 0.001).

**Figure 2:**
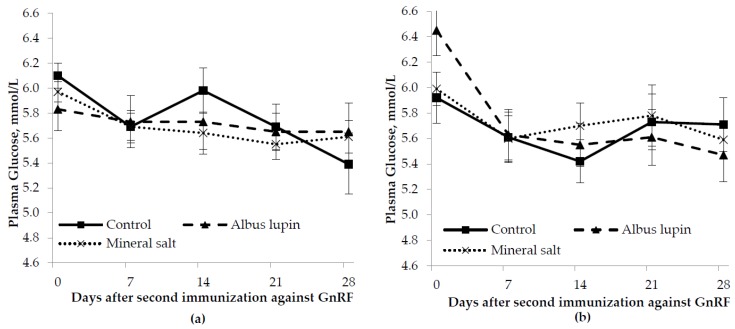
Change in plasma glucose concentration (mean ± SE) for (**a**) entire males and (**b**) immunocastrated male pigs fed three different diets for 28 days after the second immunization against gonadotrophin releasing factor (GnRF) (*n* = 12). The *p*-value for time was *p* < 0.001. All other *p*-values were not significant.

There was no effect of sex (*p* > 0.05) or diet (*p* > 0.05) however plasma glucose decreased over time (*p* < 0.001; Figure 2). There were no interactions between sex, diet and time for glucose (*p* > 0.05 for all).

**Figure 3:**
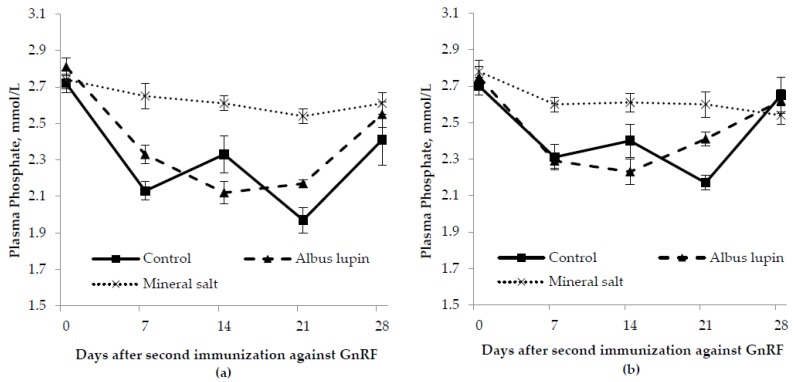
Change in plasma phosphate concentration for (**a**) entire males and (**b**) immunocastrated male pigs fed three different diets for 28 days after the second immunization against gonadotrophin releasing factor (GnRF) (*n* = 12). The *p*-values for time × diet, sex, diet and time were *p* < 0.001, *p* = 0.03, *p* < 0.001 and *p* < 0.001, respectively. All other *p*-values were not significant.

There was a time by diet interaction where pigs fed the mineral salt diet had a higher phosphate concentration compared to the albus lupin and control diet from d 7 onwards (*p* < 0.001; Figure 3). There were no other interactions (*p* > 0.05 for all). Entire male pigs had a lower plasma phosphate concentration compared to the IC males (*p* = 0.03). Pigs fed the mineral salt diet had a higher phosphate concentration compared to those fed the control and albus lupin diet (*p* < 0.001). The phosphate concentration was higher on d 0 compared to the other days (*p* < 0.001).

**Figure 4:**
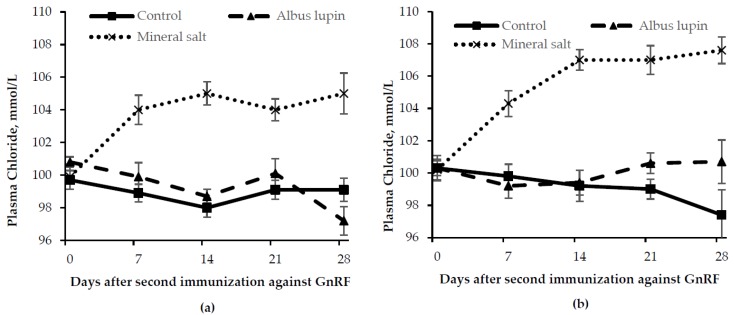
Change in plasma chloride concentration (mean ± SE) for (**a**) entire males and (**b**) immunocastrated male pigs fed three different diets for 28 days after the second immunization against gonadotrophin releasing factor (GnRF) (*n* = 12). The *p*-values for time × diet and sex were *p* < 0.001 and *p* = 0.04, respectively. All other *p*-values were not significant.

Pigs fed the mineral salt diet had an increased chloride concentration (*p* < 0.001 for time × diet interaction) from d 7 onwards compared to those fed the control or albus lupin diet (Figure 4). There were no other interactions (*p* > 0.05). Entire males had lower plasma chloride concentrations than IC males (*p* = 0.04).

**Figure 5:**
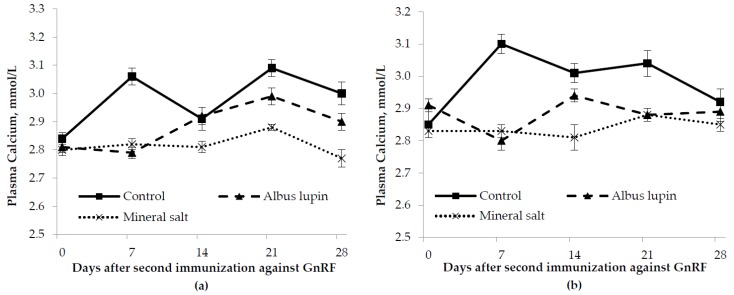
Change in plasma calcium concentration (mean ± SE) for (**a**) entire males and (**b**) immunocastrated male pigs fed three different diets for 28 days after the second immunization against gonadotrophin releasing factor (GnRF) (*n* = 12). The *p*-values for time × sex × diet, time × diet and diet were *p* = 0.05, *p* < 0.001, *p* = 0.06 and *p* < 0.001, respectively. All other *p*-values were not significant.

There was a time by sex by diet interaction in that IC male pigs fed the albus lupin and mineral salt diet had a lower calcium concentration from d 14 onwards compared to the control diet, however, only the entire males on the mineral salt diet appeared to have a lower calcium concentration from d 14 onwards. (*p* = 0.01; Figure 5). There were no other interactions (*p* > 0.05). There was no effect of sex on plasma calcium concentration (*p* > 0.05). Pigs fed the control diet had a higher calcium concentration than those on the albus diet who in turn had a higher calcium concentration than those on the mineral salt diet (*p* < 0.001). The calcium concentration was lower on d 0 compared to the other time periods (*p* < 0.001).

**Figure 6:**
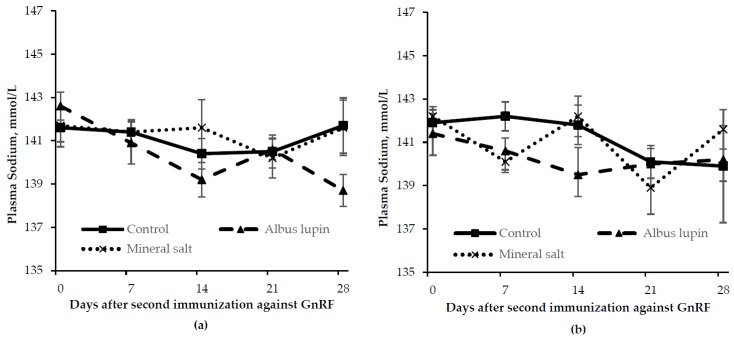
Change in sodium concentration (mean ± SE) for (**a**) entire males and (**b**) immunocastrated male pigs fed three different diets for 28 days after the second immunization against gonadotrophin releasing factor (GnRF) (*n* = 12). The *p*-value for time was *p* = 0.03. All other *p*-values were not significant.

The sodium concentration was lower (*p* = 0.03) on d 0 compared to d 14, d 21 and d 28. There was no difference in sodium concentration between d 0 and d 7 (Figure 6). There was no difference in plasma sodium concentration between sex (*p* > 0.05), or diet (*p* > 0.05) and there were no interactions (*p* > 0.05).

**Figure 7:**
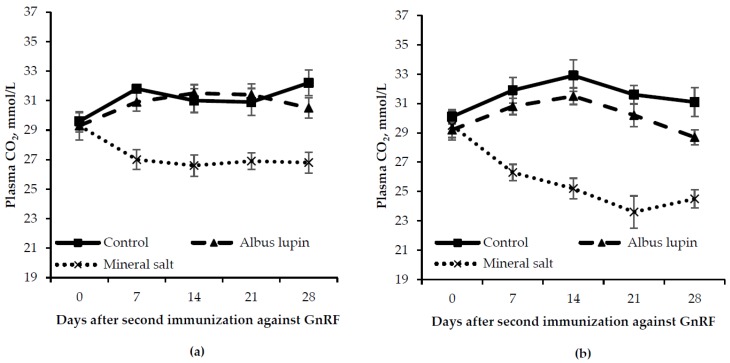
Change in plasma carbon dioxide concentration (mean ± SE) for (**a**) entire males and (**b**) immunocastrated male pigs fed three different diets for 28 days after the second immunization against gonadotrophin releasing factor (GnRF) (*n* = 12). The *p*-values for time × diet, time × sex, sex and diet were *p* < 0.001, *p* = 0.02, *p* = 0.06 and *p* < 0.001, respectively. All other *p*-values were not significant.

There was a time by diet interaction (*p* < 0.001) in that pigs fed the mineral salt diet had a lower carbon dioxide concentration from d 7 onwards compared to the other diets (Figure 7). There was also a time by sex interaction (*p* = 0.02) where on d 21 and d 28 IC males had a lower carbon dioxide concentration than entire males. There were no other interactions (>0.05). There was a trend for entire males to have a higher carbon dioxide concentration than IC males (*p* = 0.06). Pigs fed mineral salts had a lower carbon dioxide concentration compared to those fed the control or albus lupin diet (*p* < 0.001).

**Figure 8:**
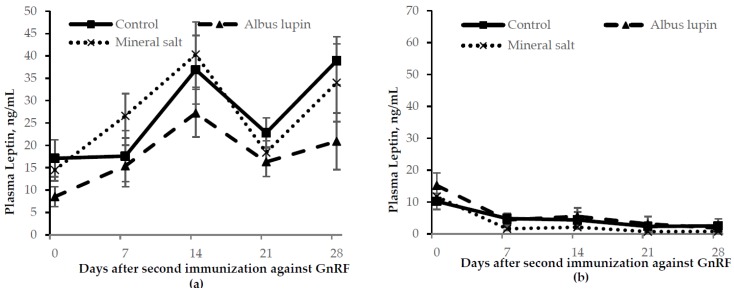
Change in plasma leptin concentration (mean ± SE) for (**a**) entire males and (**b**) immunocastrated male pigs fed three different diets for 28 days after the second immunization against gonadotrophin releasing factor (GnRF) (*n* = 12). The *p*-values for time × sex, sex × diet and sex were *p* < 0.001, *p* = 0.02 and *p* < 0.001, respectively. All other *p*-values were not significant.

There was a time by sex interaction for leptin where from d 7 onwards IC males had a lower leptin concentration compared to entire males (*p* < 0.001; Figure 8). Entire males fed the albus lupin diet had lower plasma leptin compared to those on the control or mineral salt diet (*p* = 0.02). There was no difference between diets for IC males. Entire males had a higher concentration of plasma leptin than IC males (*p* < 0.001). There were no other effects or interactions between time, sex and diet for leptin concentration (*p* > 0.05).

### 3.4. Objective Meat Quality

The objective meat quality results are given in Table 5. Pork from IC males had a higher ultimate pH (*p* < 0.001), was lighter (L*; *p* = 0.003), more yellow (*p* = 0.008) and had a higher drip loss (*p* < 0.001) compared to meat from entire males. There was no difference in relative redness, cook loss and shear force between IC males and entire males.

Pigs fed the mineral salt diet or an albus diet had a higher pH (*p* = 0.03) at 45 min post-exsanguination compared to those fed the control diet. There was no difference (*p* > 0.05) between diets for any other measure of objective meat quality. There were no interactions (*p* > 0.05) between sex and diet.

## 4. Discussion

The lysine concentrations in the diets of the entire males and the IC males were determined based on findings by Moore et al. [7]. They found that IC males show a response to dietary SID lysine similar to that of entire males for two weeks after the second immunization against GnRF. After this, IC males have a lower requirement for SID lysine than entire males. In the current experiment there was no reduction in the growth rate and feed intake of the IC males fed the control diet when the lysine concentration was reduced in the final two-week period. This indicates that the reduction in feed intake is more likely to be attributed to either the albus lupins or mineral salts and so the results have been discussed from this perspective.

The hypothesis that pigs immunized against GnRF and which were fed either a diet containing albus lupins or mineral salts would have a reduced feed intake with no effect on growth rate compared to pigs receiving a standard finisher diet was partially supported. Immunocastrated male pigs fed the mineral salt diet ate less feed than those fed the control diet with no effect on growth rate. However, IC male pigs fed the albus lupin diet had both a reduced feed intake and growth rate compared to those fed the standard finisher diet.

The results for the mineral salt diet are in contrast to Yen et al. [13] and Pluske et al. [14] who found pigs fed a mineral salt diet had both a lower daily feed intake and weight gain compared to those on the basal diet. However, Yen et al. [13] fed 4% CaCl_2_._2_H_2_O and 2.22% Na_5_P_3_O_10_ compared to the concentration of 3% CaCl_2_·2H_2_O and 1.6% Na_5_P_3_O_10_ in this experiment. Pluske et al. [14] used 4% CaCl_2_ and 2.2% Na_5_P_3_O_10_ for one week before halving the concentration for the remaining two weeks due to acceptance issues of the diet in the first week. The differences in concentration levels between studies may help to explain the observed differences in performance. In addition, the acceptance issues in Pluske et al. [14] may be because calcium chloride was used rather than calcium chloride dihydrate which increased the effective level of calcium chloride to 94% compared to 77% calcium chloride both in the current experiment and in Yen et al. [13].

The most likely explanation by which CaCl_2_ reduced feed intake is through the process of metabolic acidosis. Yen et al. [13] found that the acidosis is caused by an increase in the plasma chloride level which sets off a number of events resulting in a reduction in the buffering capacity of the HCO_3_^−^. This then leads to a low blood pH and a reduction in total CO_2_. We also found an increased plasma chloride concentration and a reduction in CO_2_. However, we did not measure blood pH or HCO_3_^−^. Although there was an increase in the dietary concentration of calcium this was not reflected in the plasma calcium concentrations because the calcium is excreted as Ca_3_(PO_4_)_2_ in the faeces [13,19]

The albus lupins were originally included in the diet at 30% based on findings from Dunshea et al. [11] and Van Nevel et al. [12]. These researchers observed reductions in feed intake in the finishing period of between 12% and 27% [11,12]. We were trying to decrease the feed intake of the IC males to similar intake levels of entire males, a reduction of approximately 15% which corresponded with the previous findings of reductions in intake. However, in the current experiment from d 0 to 7 feed intake of the pigs receiving the albus lupin diet was nearly halved compared to the control diet. It is suggested that the observed differences in intake between the experiments may be because the pigs in the ad libitum fed experiment in Dunshea et al. [11] and in van Nevel et al. [12] were acclimatized to the albus lupin diet before the experimental period. There was no acclimatization to diet in the present study.

When the daily gain, feed intake and feed conversion ratio data were examined for the d 15–28 period only and the pigs were fed a diet with 20% albus lupins, the IC male pigs on the albus lupin diet had a similar daily gain (1.05 vs. 1.09 kg/d), feed intake (3.03 vs. 3.05 kg/d) and feed conversion ratio (2.90 vs. 2.81 kg/kg) compared to entire males fed the control diet. Although there were differences in the lysine concentrations between the diets fed to IC males and entire males, the lysine concentrations were based on findings from Moore et al. [7] and were formulated to meet the requirements of the pigs at the weights and sex and were unlikely to be the factor affecting the daily gain and feed conversion. Therefore, there is potential for albus lupins to reduce the feed intake of IC males when included in the diet at 20%, however, growth rate was also reduced to similar to that of entire males.

The hypothesis that pigs fed either albus lupins or mineral salts will deposit less fat compared to pigs receiving a standard finisher (control) diet was partially supported. Irrespective of sex, pigs fed albus lupins deposited less fat compared to those on both the control and mineral salt diet. There was no difference in fat deposition between pigs fed the control or mineral salt diet. As far as we are aware there is no previous research which has investigated using these in-feed ingredients to reduce feed intake with the main objective being to reduce fat deposition.

Given that there was a reduction in feed intake in pigs receiving the mineral salt diet it was anticipated that there would be a corresponding reduction in fat deposition. However, even though there was a reduction in feed intake of 9.2% for the IC males on the mineral salt diet compared to the control diet perhaps this was insufficient to promote a decrease in fat deposition. The IC male pigs fed the mineral salt diet still consumed 9.3% more feed than the entire males fed the control diet. When diets of IC male pigs have been restricted previously by restricting the amount of feed reductions in backfat have been observed when the feed intakes were between 15% and 22% lower than pigs fed ad libitum [9].

The hypothesis that pigs immunized against GnRF and fed either a diet containing albus lupins or mineral salts would have a similar backfat compared to entire males receiving a standard finisher diet was partially supported. Immunocastrated male pigs fed the albus lupin diet had a similar backfat compared to entire males receiving the standard finisher diet. However, IC males fed the mineral salt diet were 1.36 mm fatter than entire males receiving the control diet and had a similar backfat to IC males receiving the control diet. Therefore, albus lupins show potential in reducing the increase in backfat associated with immunocastration. Further investigation should be undertaken to determine the effect of including a constant 20% albus lupins in the diet of IC males for either 28 or 14 days pre-slaughter. Van Nevel et al. [12] also found that including albus lupins in diets at 30% reduced backfat thickness with a tendency for the percentage of lean content to increase. The reduction in backfat thickness and increase in lean was attributed to the slower growth rates [12].

Plasma leptin was lower in IC males to entire males from d 7 after the second immunization against GnRF. This is in contrast with previous studies which found that plasma leptin concentrations increased in IC males compared to entire males [4,10]. Leptin is positively correlated with the amount of fat in the body [20,21]. When leptin is given endogenously there is a reduction in feed intake [22]. There is an increase in feed intake following the second immunization against GnRF and it is thought that plasma leptin increases to try to decrease feed intake to combat the increase in body fat [4]. Therefore, it was expected that plasma leptin would increase as both feed intake and fat deposition increased in IC males in the current experiment and it is not known why this was not the case. The leptin concentrations were also higher in the current experiment compared to McCauley et al. [4] and Batorek et al. [10]. This can be attributed to differences in the assay type (multi-species radioimmunoassay method versus a pig specific competitive ELISA method) and leptin specificity of the respective methods [23].

The hypothesis that feeding pigs a diet containing albus lupins or mineral salts will have no effect on objective meat quality compared to a standard finisher diet was partly supported. There was no difference in objective meat quality between any diets with the exception of pH_45 min_ which was higher in meat from pigs fed the albus lupin diet and the mineral salt diet compared to the control. When including alternative ingredients in pig diets it is important to ensure that there is no adverse impact on the meat quality of the diets. There does not appear to be any other research investigating the effect of including mineral salts in the diet on meat quality. Kim et al. [24] investigated including *Lupinus angustifolius* in pig diets at 350 g/kg and found no effect on meat quality. Although there does not appear to be any other work investigating the effect of albus lupins on meat quality in pigs, there has been some work in young bulls. When young bulls were fed diets containing either 20% albus lupins or 16.5% soybean there were no differences between diets for meat quality [25].

The IC males had an increased drip loss which is associated with the lower ultimate pH and higher L value (lighter) observed compared to entire males. This concurs with a meta-analysis by Batorek et al. [26] who found that IC males tend to have a lower ultimate pH, a higher L* value (are lighter) and an increased drip loss compared to entire males. Immunocastrated male pigs have reduced aggressive behaviour and physical activity compared to entire males and this is likely associated with the lower ultimate pH [3]. However, the meta-analysis by Batorek et al. [26] also found that IC males also have a lower shear force and no difference in the degree of yellowness compared to entire males and this was not observed in the present study.

## 5. Conclusions

A combination of calcium chloride (3%) and sodium tripolyphosphate (1.6%) can successfully be used to improve feed conversion by reducing feed intake by 9%, with no effect on growth performance in the finishing period. In this experiment, it was not effective at reducing backfat in IC male pigs. The initial inclusion concentration of albus lupins at 30% resulted in a greater reduction in feed intake than was anticipated and so the concentration was reduced after the first week. The feed intake and growth performance of IC male pigs on the albus lupin diet was similar to the entire males who received the control diet in the final two-week period at 20%. Therefore, the inclusion of albus lupins at 20% in the finisher diet also appears to have had some success in decreasing fat deposition in both entire males and IC males. Further research is warranted to investigate the effects of including albus lupins at 20% for the whole period after the second immunization against GnRF or in the second two-week period after the second immunization against GnRF only, as this is when feed intake increases considerably and when the majority of the fat deposition of IC male pigs occurs.

## Figures and Tables

**Table 1:** Composition of the experimental diet.

Ingredients g/kg, As-Fed	Control Low ^7^	Control High ^8^	Albus Lupin Low	Albus Lupin High	Mineral Salts Low	Mineral Salts High
Barley	400	400	400	400	400	400
Wheat	372	181	170	172	299	190
Mill run, 15%	50	45.7	82.3	48.5	20.0	24.6
Lupins, 28%	100	150	0	0	60	100
Lupins albus	0	0	300	300	0	0
Canola meal, 36%	0	150	0	0	100	150
Soybean meal, 48%	10	10	0	19.5	10	10
Bloodmeal, 85%	12.6	8.81	0	20	4.15	19.1
Tallow	36.0	38.4	30.4	21.9	55	55
Limestone	12.2	10.3	11.3	11.1	0	0
Salt	2.00	2.00	2.00	2.00	2.00	2.00
L-Lysine HCL	2.13	2.12	0.22	0.42	1.98	1.96
Methionine	0.61	0.51	0.16	1.01	0	0.51
Phytase ^1^	0.20	0.20	0.20	0.20	0.20	0.20
Choline chloride, 60%	1.24	0	2.05	1.93	0.47	0
Calcium chloride, 77% ^2^	0	0	0	0	16.0	16.0
Sodium tripolyphosphate ^3^	0	0	0	0	3.00	3.00
Vitamins and minerals ^4^	1.00	1.00	1.00	1.00	1.00	1.00
Nutrient Composition ^5^						
DE, MJ/kg	14.0	14.0	14.0	14.0	14.0	14.0
CP, g/kg	145	188	184	207	150	181
Ca, g/kg	8.00	8.00	8.00	8.00	12.2	12.5
Total P, g/kg	5.92	6.83	6.40	6.24	10.2	10.6
Available P, g/kg	4.22	4.50	4.50	4.50	7.74	7.86
Na, g/kg	1.09	1.15	0.96	1.10	6.02	6.18
NDF, g/kg	16.8	19.7	18.9	17.8	16.6	17.8
ADF, g/kg	5.30	5.63	7.00	7.10	4.34	4.74
g SID Lysine/MJ DE ^6^	0.50	0.64	0.50	0.64	0.50	0.64

^1^ Phytase from Phyzyme, Danisco Australia Pty Ltd., Botany, NSW, Australia; ^2^ Calcium chloride dihydrate, Redox Pty Ltd., Bibra Lake, WA, Australia; ^3^ Sodium tripolyphosphate STPP FCC Low Nitrate/Nitrite, Redox Pty Ltd. Bibra Lake, WA, Australia; ^4^ Provided per kg of final diet: 7000 IU Vitamin A, 1400 IU Vitamin D3, 20 g Vitamin E, 1 g Vitamin K, 1 g Vitamin B1, 3 g Vitamin B2, 1.5 g Vitamin B6, 15 mg Vitamin B12 12 g niacin, 10 mg pantothentic acid, 0.19 g folic acid, 30 mg biotin, 10.6 g Calcium pantothenatic, 60 g iron, 100 g zinc, 40 g manganese, 10 g copper, 0.2 g cobalt, 0.5 g iodine, 0.3 g selenium, and 20 g antioxidant; ^5^ Calculated composition; ^6^ SID—standard ileal digestible; ^7^ Low—lysine concentration was 0.50 g standard ileal digestible lysine/MJ DE; ^8^ High—Lysine concentration was 0.64 g standard ileal digestible lysine/MJ DE.

**Table 2:** Quantitative amino acid analysis of the diets.

Amino acid g/kg, As-Fed	Control Low ^1^	Control High ^2^	Albus Lupins Low	Albus Lupins High	Mineral Salts Low	Mineral Salts High
Histidine	3.9	4.4	3.8	4.9	3.5	5.0
Isoleucine	5.1	6.0	6.2	6.7	5.1	5.9
Leucine	10.3	11.5	11.2	14.0	9.6	12.9
Lysine	8.1	9.5	7.4	9.4	7.7	10.3
Methionine	2.6	2.5	1.6	2.2	1.9	1.2
Phenylalanine	6.5	7.1	6.8	8.3	6.1	7.8
Threonine	5.5	6.3	5.8	6.9	5.2	6.7
Valine	7.4	8.3	7.4	9.2	6.9	9.2
Alanine	6.4	7.0	6.1	7.5	5.9	7.6
Arginine	9.4	10.8	12.9	14.8	8.2	10.8
Aspartic acid	10.9	12.4	13.4	15.9	10.0	13.3
Glycine	7.1	7.8	7.0	7.8	6.6	7.6
Glutamic acid	27.8	30.4	32.1	35.0	26.5	30.9
Proline	9.6	10.2	9.5	10.2	9.5	10.2
Serine	6.7	7.5	7.7	9.0	6.1	7.9
Tyrosine	3.4	3.8	4.2	4.8	3.0	3.6

^1^ Low—lysine concentration was 0.50 g standard ileal digestible lysine/MJ DE; ^2^ High—Lysine concentration was 0.64 g standard ileal digestible lysine/MJ DE.

**Table 3:** Growth and carcass performance for entire male and immunocastrated male pigs fed three different diets from 67.5 to 95.4 kg liveweight (*n* = 7).

	Entire Male			Immunocastrated Male			SED ^a^	*p*-Value		
	Control	Mineral Salt	Albus Lupin	Control	Mineral Salt	Albus Lupin		Sex	Diet	Sex × Diet
**Daily gain (kg/day)**										
d 0–14	1.00	0.984	0.648	1.07	1.00	0.673	0.037	0.11	<0.001	0.67
d 15–28	1.09	1.06	0.930	1.23	1.25	1.050	0.036	<0.001	<0.001	0.38
d 0–28	1.05	1.02	0.789	1.15	1.13	0.861	0.026	<0.001	<0.001	0.63
**Feed intake (kg/day)**										
d 0–14	2.52	2.25	1.61	2.63	2.39	1.64	0.076	0.04	<0.001	0.59
d 15–28	3.05	2.80	2.47	3.80	3.58	3.03	0.090	<0.001	<0.001	0.16
d 0–28	2.72	2.48	1.98	3.16	2.92	2.30	0.075	<0.001	<0.001	0.46
**Feed conversion ratio (kg/kg)**										
d 0–14	2.52	2.29	2.51	2.48	2.39	2.45	0.094	0.98	0.05	0.45
d 15–28	2.81	2.64	2.67	3.10	2.86	2.90	0.091	<0.001	0.007	0.82
d 0–28	2.60	2.43	2.52	2.75	2.59	2.67	0.058	<0.001	0.001	0.98
**Carcass characteristics**										
CW ^c^ (kg)	64.7	64.3	58.8	65.6	65.2	59.8	0.820	0.06	<0.001	1.00
DP ^d^ (%)	66.8	66.9	65.7	65.9	65.6	65.1	0.428	<0.001	0.01	0.40
P2 backfat (mm) ^b^	9.34	8.83	7.49	10.8	10.7	8.71	0.508	<0.001	0.01	0.43

^a^ Standard Error Difference for Sex×Diet; ^b^ Carcass weight used as a covariate; ^c^ Carcass weight; ^d^ Dressing percentage.

**Table 4:** Body composition for entire male and immunocastrated male pigs fed three different diets from 67.5 to 95.4 kg liveweight (*n* = 12).

	Entire Male			Immunocastrated Male			SED ^a^	*p*-Value		
	Control	Mineral Salt	Albus Lupin	Control	Mineral Salt	Albus Lupin		Sex	Diet	Sex × Diet
**BMC ^b^ (g/day)**										
d −1–13	22.0	20.6	13.4	23.3	23.1	16.9	1.94	0.03	<0.001	0.73
d 14–27	18.1	23.9	20.5	16.5	20.5	17.1	2.00	0.02	0.003	0.76
d −1–27	20.2	22.3	16.9	19.6	22.7	17.0	1.39	0.98	<0.001	0.88
**Lean (g/day)**										
d −1–13	736	709	499	783	694	525	43.2	0.446	<0.001	0.59
d 14–27	706	765	752	737	762	626	52.5	0.29	0.14	0.10
d −1–27	725	737	625	758	728	576	34.1	0.67	<0.001	0.24
**Fat (g/day)**										
d −1–13	189	196	57.6	202	173	97.9	30.9	0.57	<0.001	0.35
d 14–27	238	294	166	465	487	369	38.5	<0.001	<0.001	0.81
d −1–27	222	245	112	321	330	234	23.2	<0.001	<0.001	0.53
**g Fat/kg Metabolic liveweight**										
d −1–13	423	399	160	405	350	261	70.6	0.78	<0.001	0.29
d 14–27	445	551	367	770	788	712	66.0	<0.001	0.03	0.47
d −1–27	266	281	170	344	353	304	25.6	<0.001	<0.001	0.18

^a^ Standard Error Difference for Sex × Diet; ^b^ BMC—bone mineral content.

**Table 5:** Objective meat quality for entire male and immunocastrated male pigs fed three different diets (*n* = 21).

	Entire Male			Immunocastrated Male			SED ^a^	*p*-Value		
	Control	Mineral Salt	Albus Lupin	Control	Mineral Salt	Albus Lupin		Sex	Diet	Sex × Diet
pH 45 min	6.18	6.24	6.24	6.02	6.26	6.20	0.082	0.23	0.03	0.32
pH 24 h	5.62	5.54	5.62	5.58	5.67	5.52	0.043	<0.001	0.81	0.22
L	49.4	48.7	48.5	50.8	50.4	51.1	1.10	0.003	0.77	0.69
a	5.82	5.56	5.08	6.01	5.31	6.04	0.377	0.17	0.17	0.08
b	4.15	3.91	3.51	4.54	4.16	4.64	0.377	0.01	0.46	0.21
Drip loss (%)	4.75	3.79	3.57	6.12	5.04	6.18	0.725	<0.001	0.14	0.34
Cook loss (%) ^b^	24.5	23.6	23.6	21.9	23.1	25.8	1.14	0.73	0.35	0.12
Shear force (N) ^b^	47.0	42.6	45.1	41.3	43.4	44.7	2.71	0.41	0.78	0.43

^a^ Standard Error Difference for Sex × Diet; ^b^ For Batch 1 only (*n* = 12). Batch 2 samples were accidentally aged.
